# Targeting MCL-1 sensitizes human esophageal squamous cell carcinoma cells to cisplatin-induced apoptosis

**DOI:** 10.1186/s12885-017-3442-y

**Published:** 2017-06-28

**Authors:** Xinfang Yu, Wei Li, Zhenkun Xia, Li Xie, Xiaolong Ma, Qi Liang, Lijun Liu, Jian Wang, Xinmin Zhou, Yifeng Yang, Haidan Liu

**Affiliations:** 1 Clinical Center for Gene Diagnosis and Therapy, The Second Xiangya Hospital of Central South University, 139 Renmin Road, Changsha, Hunan 410011 China; 2 Department of Cardiovascular Surgery, The Second Xiangya Hospital of Central South University, 139 Renmin Road, Changsha, Hunan 410011 China; 3 Hunan Cancer Hospital and The Affiliated Cancer Hospital of Xiangya School of Medicine, Central South University, 283 Tongzipo Road, Changsha, Hunan 410013 China; 4 Department of Radiology, The Third Xiangya Hospital of Central South University, 138 Tongzipo Road, Changsha, Hunan 410013 China; 5 Department of Thoracic Surgery, The Second Xiangya Hospital of Central South University, 139 Renmin Road, Changsha, Hunan 410011 China

**Keywords:** Esophageal squamous cell carcinoma, Chemosensitization, MCL-1, Cisplatin, Apopotosis

## Abstract

**Background:**

Esophageal squamous cell carcinoma (ESCC) is one of the most lethal malignancies in China and is an exceptionally drug-resistant tumor with a 5-year survival rate less than 15%. Cisplatin is the most commonly used conventional chemotherapeutic drug for the treatment of ESCC, but some patients have a poor response to cisplatin-based chemotherapy. New strategies that could enhance chemosensitivity to cisplatin are needed.

**Methods:**

We used reverse transcription-RCR (RT-PCR), immunoblot, immunohistochemical (IHC) staining, anchorage-dependent and -independent growth assays, co-immunoprecipitation (Co-IP) assay, RNA interference and in vivo tumor growth assay to study the expression of MCL-1 in ESCCs and the response of ESCC cells to cisplatin.

**Results:**

The present study showed that MCL-1 expression was significantly increased in ESCC tissues compared to normal adjacent tissues and was associated with depth of invasion and lymph node metastasis. Knockdown of MCL-1 produced significant chemosensitization to cisplatin in association with caspase-3 activation and PARP cleavage in KYSE150 and KYSE510 cells. The selective MCL-1 inhibitor UMI-77 caused dissociation of MCL-1 from the proapoptotic protein BAX and BAK, and enhanced KYSE150 and KYSE510 cells to cisplatin-induced apoptosis accompanied by caspase-3 activation and PARP cleavage.

**Conclusions:**

The current study suggests that MCL-1 contributes to the development of ESCC and is a promising therapeutic target for chemosensitization of ESCC cells to cisplatin. This might provide a scientific basis for developing effective approaches to treat the subset of ESCCs patients with MCL-1 overexpression.

## Background

Esophageal squamous cell carcinoma (ESCC) is one of the most frequently diagnosed cancers in developing countries, especially in Eastern Asia [1]. Although the therapy strategies have been improved, ESCC is still one of the most aggressive types of cancer with poor prognosis and rapid progression and the 5-year overall survival rate less than 15%. Palliative chemotherapy with platinum-based regimen is generally indicated for patients with ESCC. Response rate to these platinum drugs is modest. Hence, there is a need for a novel treatment modality involving these anticancer drugs that selectively target cancer cells and circumvent treatment-resistant pathways for the management of ESCC.

Myeloid cell leukemia 1 (MCL-1) is a major prosurvival member of the Bcl-2 family proteins, which is mainly localized to the outer mitochondrial membrane via its C-terminal transmembrane (TM) domain [2]. Studies demonstrated the MCL-1 is important for cell proliferation, differentiation and tumorigenesis by regulating the apoptosis pathway [3, 4]. By sequestering the proapoptotic multidomain proteins BAX and BAK, MCL-1 inhibits permeabilization of the mitochondrial membrane and ultimately preventing apoptosis. MCL-1 is subject to negative regulation by BH3-only protein family (e.g. NOXA, BIM and PUMA), which specifically bind to the BH3 binding groove, formed by BH domains of MCL-1, displacing MCL-1 from BAX and/or BAK and thus promoting apoptosis. MCL-1 frequently overexpressed in a variety of human tumor tissues, including stomach [3], liver [5], pancreas [6], prostate [7] and lung [8], which contributes to tumor development and progression and associated with poor patient prognosis. High expression of MCL-1 has been shown in human esophageal carcinoma cell lines including CE8 1 T/VGH, KYSE450, TE-1, Eca109, KYSE150 and KYSE510 [9–11]. However, whether MCL-1 is overexpressed in human primary ESCC tumors and contributes to ESCC development and progression remains unclear.

Cisplatin is frequently used for the treatment of various cancers, including ESCC, but some patients have a poor response to cisplatin-based chemotherapy. New strategies that could enhance chemosensitivity to cisplatin are needed. Overexpression of MCL-1 is frequently resistance to various cancer therapies, including chemotherapy [12]. Genetic silencing of *Mcl-1* sensitizes a spectrum of cancers, such as melanoma, non-small cell lung and hepatocellular cancers to chemotherapy [8]. In addition, plenty of researches showed that the expression level of MCL-1 determines the sensitivity of multiple cancers to cisplatin. For instance, microRNA-193b enhances the cytotoxicity of cisplatin to hepatocellular carcinoma cells by targeting *Mcl-1* [13]. Imperatorin acts as a cisplatin sensitizer via down-regulating MCL-1 expression in HCC chemotherapy [14]. The selective Wee-1 kinase inhibitor AZD-1775 sensitizes HPV-positive HNSCC cells to cisplatin-induced apoptosis in vitro accompanied by selective decrease in expression of MCL-1 and XIAP antiapoptotic proteins [15]. Knockdown of MCL-1 by siRNA or inhibition of MCL-1 by specific pharmacologic inhibitor EU-5148, sensitizes TWEAK-treated non-small cell lung cancer cells to cisplatin-mediated apoptosis [8]. Knockdown of MCL-1 also enhances sensitivity to cisplatin in gastric cancer cells expressing high levels of MCL-1 [16]. Considering high expression of MCL-1 in some ESCC cell lines [9–11], MCL-1 might function as an effective target to enhance the sensitivity of ESCC cells to cisplatin. However, whether MCL-1 inhibition acts as a cisplatin-chemosensitizing strategy in ESCC cells and the underlying mechanism remains incompletely defined.

In the current study, we found that MCL-1 expression was significantly increased in ESCC tissues compared to normal adjacent tissues and was associated with depth of invasion and lymph node metastasis. Moreover, MCL-1 inhibition by either genetical or pharmacological approach significantly enhanced the cytotoxicity of cisplatin to ESCC cells. The combination of UMI-77 and cisplatin induced apoptosis more significantly compared with treatment of UMI-77 or cisplatin alone by causing caspase-3 activation and PARP cleavage. In addition, the results demonstrated that UMI-77 prevented MCL-1/BAX and MCL-1/BAK complexes formation. To our knowledge, this is the first report to demonstrate that the chemosensitizing effect of a selective MCL-1 inhibitor UMI-77 combined with cisplatin to treat ESCC cells. The results suggested that MCL-1 is a promising therapeutic target for chemosensitization of ESCC cells to cisplatin and might provide a scientific basis for developing effective approaches to treatment human ESCCs.

## Methods

### Clinical tissue sample collections

Fresh tumor tissues and the corresponding normal adjacent tissues of the same patient with pathologically and clinically confirmed ESCC were collected from 49 patients by the Department of Cardiothoracic Surgery, The Second Xiangya Hospital of Central South University, Changsha, Hunan, China. Several small pieces of fresh tumor tissue samples were dissected from the main tumor part of each surgically removed specimen. A portion of tumor and normal adjacent tissues were frozen immediately in liquid nitrogen and then stored at −80 °C for protein and mRNA extraction and analysis of MCL-1 expression by RT-PCR and Western blotting, respectively. A portion of tumor and normal adjacent tissues were fixed in formalin solution and sent for histological examination. The paraffin-embedded sections from the specimens, which were diagnosed as having ESCC, were used for immunostaining of MCL-1 protein expression. All tumors were confirmed as ESCC by the Clinicopathologic Department at the Second Xiangya Hospital of Central South University. All cases were classified according to the sixth edition of the pathologic tumor-node-metastasis (pTNM) classification. All the patients received no treatment before surgery.

### Cell lines and culture

The KYSE150, KYSE510, Eca109, and TE-1 ESCC cell lines were obtained and grown in RPMI-1640 medium supplemented with 10% FBS and 1% antibiotics as previously reported [9]. Het-1A, a non-tumourigenic SV40T-immortalized human esophageal epithelial cell line [17], was purchased from the American Type Culture Collection (Manassas, VA). The 293 T cell line was obtained as previously reported [18]. Het-1A and 293 T cells were cultured with Dulbecco’s Modified Eagle Medium (DMEM) containing 10% FBS and 1% antibiotics. All cell lines were incubated at 37 °C in a humidified atmosphere containing 5% CO_2_. Each vial of frozen cells was thawed and maintained for 2 months (10 passages). The cells were cultured for 36 to 48 h and proteins extracted for analysis.

### Reagents

Chemical reagents, including Tris, NaCl, and SDS, for molecular biology and buffer preparation were purchased from Sigma-Aldrich (St. Louis, MO). UMI-77 (Cat. No. S7531, Selleck Chemicals) was dissolved in DMSO at 100 mM and stored in aliquot at −80 °C. Aliquots were diluted in corresponding medium just before addition to cell cultures. Cisplatin (Cat. No. 479306) was purchased from Sigma-Aldrich (St. Louis, MO). Stock cisplatin solution was prepared in DMSO at 200 mM stored as aliquots at −80 °C and used within 1 week and further diluted in medium before adding to the cells. The short hairpin RNAs (shRNAs) against human *Mcl-1* were purchased from Thermo Scientific. Two targeting sequences, *pLKO.1-shMCL-1#1*, CCGGGCTAAACACTTGAAGACCATACTCGAGTATGGTCTTCAAGTGTTTAGCTTTTTG and *pLKO.1-shMCL-1#2,* CCGGGCAGAAAGTATCACAGACGTTCTCGAGAACGTCTGTGATACTTTCTGCTTTTTG, were used in the study. *pLKO.1-shGFP* (plasmid #30323), the lentiviral packaging plasmid *psPAX2* (plasmid #12260) and the envelope plasmid *pMD2.G* (plasmid #12259) were available on Addgene (Cambridge, MA).

### Cell proliferation assays

Cell proliferation assays were performed as previously described [18].

### Anchorage-independent cell growth assays

Cells (8 × 10^3^ per well) were seeded into 6-well plates with 0.3% Basal Medium Eagle agar containing 10% FBS and cultured. The cultures were maintained at 37 °C in a 5% CO_2_ incubator for 2 or 3 weeks and colonies were counted under a microscope as previously described [18]*.*

### Protein preparation and Western blot analysis

Frozen tissue samples were sectioned into small pieces and dissolved in lysis buffer containing 50 mM Tris-Cl (pH 8.0), 150 mM NaCl, 0.1% SDS, 100 μg/ml phenylmethylsulfonyl fluoride, 2 μg/ml aprotinin, 2 μg/ml leupeptin, 1% NP-40. The samples were homogenized, sonicated and kept on ice for 30 min. After centrifugation, the supernatant was collected for immunoblotting analysis. Cultured cells were harvested and whole cell lysates were prepared according to the method previously described [18]. Protein concentration was determined using the BCA Assay Reagent (Cat. no. 23228, Pierce, Rockford, IL). Western blotting was performed as previously described [18]. Primary antibodies were used for immunoblotting: MCL-1 (#5453), cleaved caspase-3 (#9664), cleaved PARP (#5625), BCL-2 (#2870), BCL-xL (#2764), BAX (#5023) and BAK (#6947) from Cell Signaling Technology; β-actin (A5316) from Sigma-Aldrich; GAPDH (sc-47,724) from Santa Cruz Biotechnology. Secondary antibodies were anti-rabbit IgG HRP (#7074) and anti-mouse IgG HRP (#7076) and purchased from Cell Signaling Technology. Antibody conjugates were visualized by chemiluminescence (ECL; cat#34076, Thermo).

### mRNA extraction and reverse transcription-polymerase chain reaction (RT-PCR)

Total RNA was extracted from frozen specimens using Trizol reagent (Invitrogen, Carlsbad, CA). First-strand cDNA was synthesized from 2 μg of total RNA using the Reverse Transcription System Kit (Cat. No. A3500, Promega, Madison, WI). The resulted cDNA was subjected to PCR (95 °C for 5 min followed by 36 cycles of 95 °C for 30 s, 55 °C for 30 s, 72 °C for 40 s, and an extension for 10 min at 72 °C) using primers designed for human *Mcl-1* [9]: sense, 5′-cggcagtcgctggagattat-3′ and antisense, 5′-gtggtggtggttggtta-3′, yield a 573-bp product; or for *β-actin*: sense, 5′-ttccagccttccttcctggg-3′ and antisense, 5′-ttgcgctcaggaggagcaat-3′, yield a 224-bp product. PCR products were separated on 1.5% agarose gels and visualized with ethidium bromide.

### Immunohistochemical (IHC) staining

Tumor tissues obtained from ESCC patients or euthanized xenografted mice were embedded in paraffin and subjected to immunohistochemistry staining with specific antibodies against MCL-1 (1:100, sc-819, Santa Cruz Biotechnology) or Ki67 (1:200, ab16667, Abcam) according to the DAKO system protocol. Hematoxylin was used for counterstaining. Slides were viewed and photographed under a light microscope, and analyzed using Image-Pro Plus Software (version 6.2) program (Media Cybernetics).

### RNA interference

The generation of gene stable knockdown cell lines was performed as described previously [18]. Briefly, to generate MCL-1 knocking down cells, *pLKO.1-shGFP*, *pLKO.1-shMCL-1#1* and *pLKO.1-shMCL-1#2* lentivirus plasmids were cotransfected into 293 T cells with *pSPAX2* and *pMD2.G*. Viral supernatant fractions were collected at 48 h after transfection and filtered through a 0.45 μm filter followed by infection into KYSE150 and KYSE510 cells together with 6 μg/mL polybrene. At 16 h after infection, the medium was replaced with fresh medium containing 2 μg/mL puromycin and cells were incubated for another 6 days. For transient knockdown MCL-1, KYSE150 and KYSE510 cells were grown in 6-well plates and transfected with an *Mcl-1* siRNA (Cat. No. SC-35877; Santa Cruz Biotechnology) or a control siRNA (Cat no. sc-37,007; Santa Cruz Biotechnology) using HiPerFect transfection reagent (Cat no. 301705, Qiagen) for 48 h according to the manufacturer’s instructions. Cells were then harvested for protein extraction and immunoblotting to confirm MCL-1 knockdown.

### Co-immunoprecipitation (co-IP) assays

Co-IP assays were performed as described previously [19]. Briefly, cells were serum-starved in 0.1% FBS/RPMI 1640 medium overnight followed by treatment with DMSO or the indicated concentrations of UMI-77 for 48 h. Cells were harvested in IP lysis buffer (Cat. No. 87788, Thermo Scientific) as described by the manufacturer. Cell lysates were pre-cleared with 40 μl of protein A/G-agarose beads (sc-2003, Santa Cruz Biotechnology) and immunoprecipitated with 2 μg of anti-MCL-1 (sc-819, Santa Cruz Biotechnology) or normal rabbit IgG (NI01, Calbiochem) at 4 °C overnight, followed by 2 h of incubation at 4 °C with 40 μl of protein A/G-agarose beads. Immunocomplexes were resolved by SDS-PAGE and co-immunoprecipitated proteins were detected using anti-BAX (#5023, Cell Signaling Technology) and anti-BAK (#6947, Cell Signaling Technology) antibodies, respectively.

### In vivo tumor growth assay

All mouse studies were performed utilizing protocols approved by the Institutional Animal Care and Use Committee of the Second Xiangya Hospital of Central South University. Athymic nude mice (BALB/c nude mouse, 6 wk. old) were randomly divided into two groups (*n* = 10) and subcutaneously injected in the flank with KYSE150-shGFP or KYSE150-shMCL-1#2 esophageal carcinoma cells (2 × 10^6^). Mice were weighed and tumors measured by caliper every other day. Tumor volume was calculated from measurements of 2 diameters of the individual tumor according to the following formula: tumor volume (mm^3^) = (length × width × width/2) [19]. Mice were monitored until day 27 and at that time mice were euthanized and tumors extracted.

### Statistical analysis

Statistical analysis was done with the statistical software program SPSS ver.12.0. The statistical significance of the correlations between MCL-1 overexpression and clinicopathologic characteristics were assessed by *χ* ^*2*^ test or Fisher’s exact test. Results expressed as mean ± SD were analyzed using the Student’s *t* test. Differences were considered significant when *p* < 0.05.

## Results

### MCL-1 is overexpressed in human esophageal squamous cell carcinoma

**Fig. 1:**
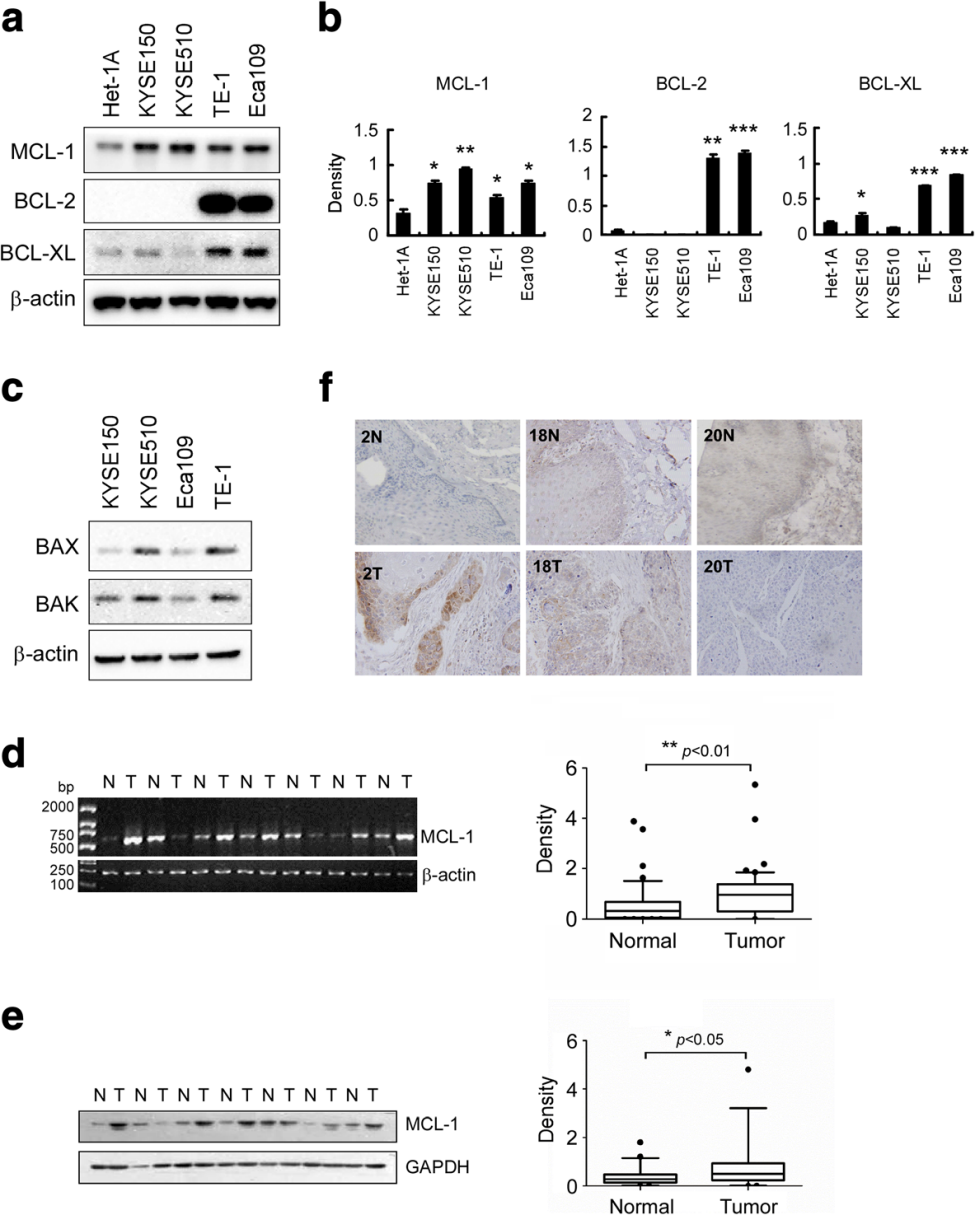
MCL-1 is overexpressed in human esophageal squamous cell carcinoma. **a**, **b** Western blot analysis was performed to examine MCL-1, BCL-2 and BCL-xL expression in several ESCC cell lines and normal Het-1A cells. β-actin was used as a loading control (**a**). The density of each protein was shown (**b**). The intensity was evaluated using Image J (NIH) computer software. *p < 0.05, **p < 0.01, ***p < 0.001, significant difference compared with the Het-1A cells. **c** Western blot analysis was performed to examine BAX and BAK expression in several ESCC cell lines. β-actin was used as a loading control. **d** Representative results of RT-PCR analysis showed *Mcl-1* mRNA levels in ESCC tumors (T) compared with normal adjacent epithelia tissues (N). β-actin was used as a loading control (*left panel*). The levels of *Mcl-1* mRNA in malignant and the corresponding normal adjacent tissues of 49 ESCC patients were shown (*right panel*). The intensity was evaluated using Image J (NIH) computer software. **, *p* < 0.01, significant difference between groups as indicated. **e** Representative results of Western blot analysis showed MCL-1 protein levels in malignant tissues (T) and the corresponding normal adjacent tissue samples (N). GAPDH was used as a loading control (*left panel*). The levels of MCL-1 protein in malignant and the corresponding normal adjacent tissues of 49 ESCC patients were shown (*right panel*). The intensity was evaluated using Image J (NIH) computer software. *, *p* < 0.05, significant difference between groups as indicated. **f** Representative results of immunohistochemistry staining showed up-regulation (cases 2), no significant change (case 18) and down-regulation (case 20) of MCL-1 protein in ESCC tumors (T) compared with the corresponding normal adjacent tissues (N) (original magnification, ×200)

To investigate whether the high expression of MCL-1 is linked to human esophageal squamous cell carcinoma, expression of MCL-1 was examined. When compared to a non-tumourigenic squamous esophageal Het-1A cell line, MCL-1 was highly expressed in all ESCC cell lines tested **(**Fig. 1a and b **)**. Expression of prosurvival proteins BCL-2 and BCL-xL were also examined. Results showed that no expression of BCL-2 in KYSE150 and KYSE510 cells. BCL-xL was expressed in all ESCC cell lines evaluated with barely detectable amount in KYSE510 and relatively low expression in KYSE150 cells **(**Fig. 1a and b **)**, suggesting these two cell lines depend mainly on MCL-1 for survival. We therefore selected KYSE150 and KYSE510 cell lines for further investigation. In addition, expression of BAX and BAK proteins in these four ESCC cell lines were estimated **(**Fig. 1c **)**. We next sought to examine the expression levels of *Mcl-1* mRNA and protein in ESCCs and the matched normal adjacent tissue specimens. Thirty-one tumors of 49 cases (63.3%) showed elevated *Mcl-1* mRNA and protein levels compared to the paired normal tissues. The difference of MCL-1 overexpression between the tumor and the normal groups was statistically significant (Fig. 1d and e). In addition, immunohistochemical (IHC) approach was also used to examine MCL-1 expression. Representative results showed increased, equal or decreased level of MCL-1 in human ESCC tumors and the matched normal adjacent tissues **(**Fig. 1f **)**. The IHC results were in agreement with the observations by RT-PCR and immunoblotting analysis shown above. These results implicate that MCL-1 might be a critical molecule in human esophageal squamous cell carcinoma development.

### Relationship between MCL-1 overexpression and clinicopathological parameters in patients with ESCC

The association between MCL-1 overexpression and clinical features of patients, including age, gender, tumor location, tumor size, histopathologic characteristics, initial clinical stage, depth of invasion, and lymph node status were summarized in Table 1. MCL-1 overexpression was positively correlated with depth of invasion (*p* = 0.0131, Table 1). In addition, the frequency of present MCL-1 overexpression was significantly higher in ESCCs with lymph node metastasis than those without metastasis (*p* = 0.0174, Table 1). No significant association was observed between MCL-1 overexpression and other clinicopathological variables, including tumor size, tumour differentiation, and other features. Since MCL-1 functions as a potent inhibitor of apoptosis and its aberrant results in impaired apoptosis [20, 21], our observations was in agreement with a previous report that impaired apoptosis would cause abnormal cell growth and lead to certain malignant phenotypes, such as tumor invasion and metastasis [22]. The results suggest that MCL-1 overexpression plays a significant role in malignant progression of ESCC.

**Table 1:** Relationships between MCL-1 overexpression and clinicopathologic characteristics in patients with esophageal squamous cell carcinoma

		MCL-1		*p–*value
Characteristics	All cases	Non-overexpression	Over-expression	
Gender				1.0000
Male	47	17	30	
Female	2	1	1	
Age (years)				0.2436
≤ 60	25	7	18	
> 60	24	11	13	
Location				0.4568
Upper	4	2	2	
Middle	26	7	19	
Lower	19	8	11	
Initial clinical stage				0.2168
≤ IIa	16	8	8	
> IIa	33	10	23	
Tumor size				0.3309
< 5 cm	13	6	7	
≥ 5 cm	36	11	25	
Differentiation				0.3262
Poorly	16	6	10	
Moderately	18	4	14	
Well	15	7	8	
Depth of invasion				0.0131*
Submucosa	5	2	3	
Muscularis	28	14	14	
Adventia	16	1	15	
Nodal metastasis				0.0174*
Absent	20	11	9	
Present	29	6	23	

*p*-value was calculated using Fisher’s exact test or Pearson’s χ^2^ test. *p < 0.05 indicates a significant association among the variables

### Knockdown of MCL-1 inhibits the proliferation of esophageal squamous cell carcinoma cells in vitro

**Fig. 2:**
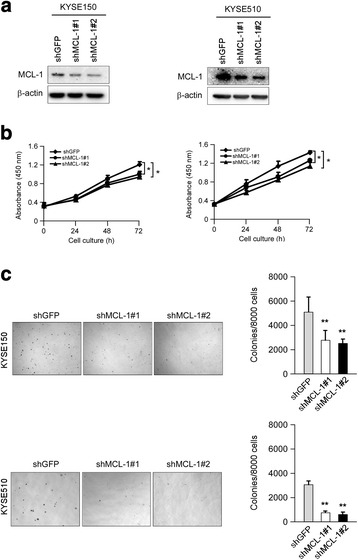
Stable knockdown of MCL-1 expression reduces ESCC cells proliferation in vitro. **a** Validation of the MCL-1 knockdown by shRNAs in ESCC cells. KYSE150 and KYSE510 cells were transduced with lentiviral short hairpin RNAs targeting GFP (shGFP) and MCL-1 (shMCL-1#1 and #2). The knockdown of MCL-1 expression was confirmed by Western blot analysis 3 d after shRNAs transduction. β-actin was used as a loading control. **b** Knockdown of MCL-1 attenuates KYSE150 and KYSE510 anchorage-dependent cell growth. WST-1 assays were performed as described in Materials and Methods. All experiments were performed in triplicate with at least two independent experiments. *p < 0.05, significant difference compared with the shGFP cells at 72 h. **c** Knockdown of MCL-1 attenuates KYSE150 and KYSE510 anchorage-independent cell growth. Soft agar assays were performed as described in Materials and Methods. **p < 0.01, significant difference in colony formation compared with the shGFP cells

**Fig. 3:**
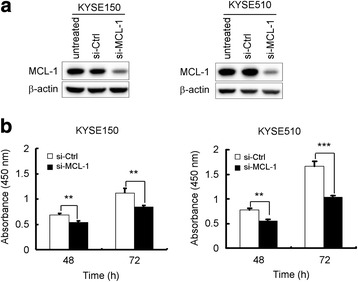
Transient reduction of MCL-1 expression in KYSE150 and KYSE510 cells suppresses cell proliferation. **a** MCL-1 protein expression was reduced by siRNA in both KYSE150 and KYSE510 cells. Cells were transiently transfected with an *Mcl-1* siRNA (si-MCL-1, 50 nM) or a control siRNA (si-Ctrl, 50 nM) for 48 h. Cell lysates were examined of MCL-1 level by Western blotting. β-actin was use as a loading control. **b** KYSE150 and KYSE510 cells were transiently transfected with an *Mcl-1* siRNA (50 nM) or a control siRNA (50 nM) for 24 h. Cell viability was measured with the WST-1 assay at 48 h and 72 h after siRNA transfection. All experiments were performed in triplicate with at least two independent experiments. **p < 0.01, ***p < 0.001, significant difference compared with the si-Ctrl cells

To examine whether knockdown of MCL-1 expression could influence the growth of ESCC cells, we generated stable KYSE150 and KYSE510 cell lines with MCL-1 knocking down. To rule out the off-target effect of the shRNA, two sequences targeting *Mcl-1* gene were used and validated effective attenuation of MCL-1 expression **(**Fig. 2a **)**. WST-1 assays showed that both KYSE150 and KYSE510 cells expressing shRNA targeting *Mcl-1* suppressed cell proliferation **(**Fig. 2b **)**. To further investigate whether MCL-1 affects the tumorigenic properties in ESCC, we measured anchorage-independent growth of these two stable KYSE150 and KYSE510 cell lines. Knockdown of MCL-1 expression significantly attenuated the colony formation of these cell lines in soft agar **(**Fig. 2c **)**. In addition, we used an *Mcl-1*-specific small interfering RNA oligonucleotide to silence endogenous MCL-1 expression. The *Mcl-1* siRNA led to a considerable knockdown of MCL-1 protein expression **(**Fig. 3a **)** and caused more potent cell proliferation inhibitory effects **(**Fig. 3b **)** in both KYSE150 and KYSE510 cells. The results demonstrate that diminishment of MCL-1 expression significantly reduces the tumorigenic properties of esophageal squamous cancer cells in vitro.

### Knockdown of MCL-1 inhibits the growth of esophageal squamous cell carcinoma cells in a xenograft mouse model

**Fig. 4:**
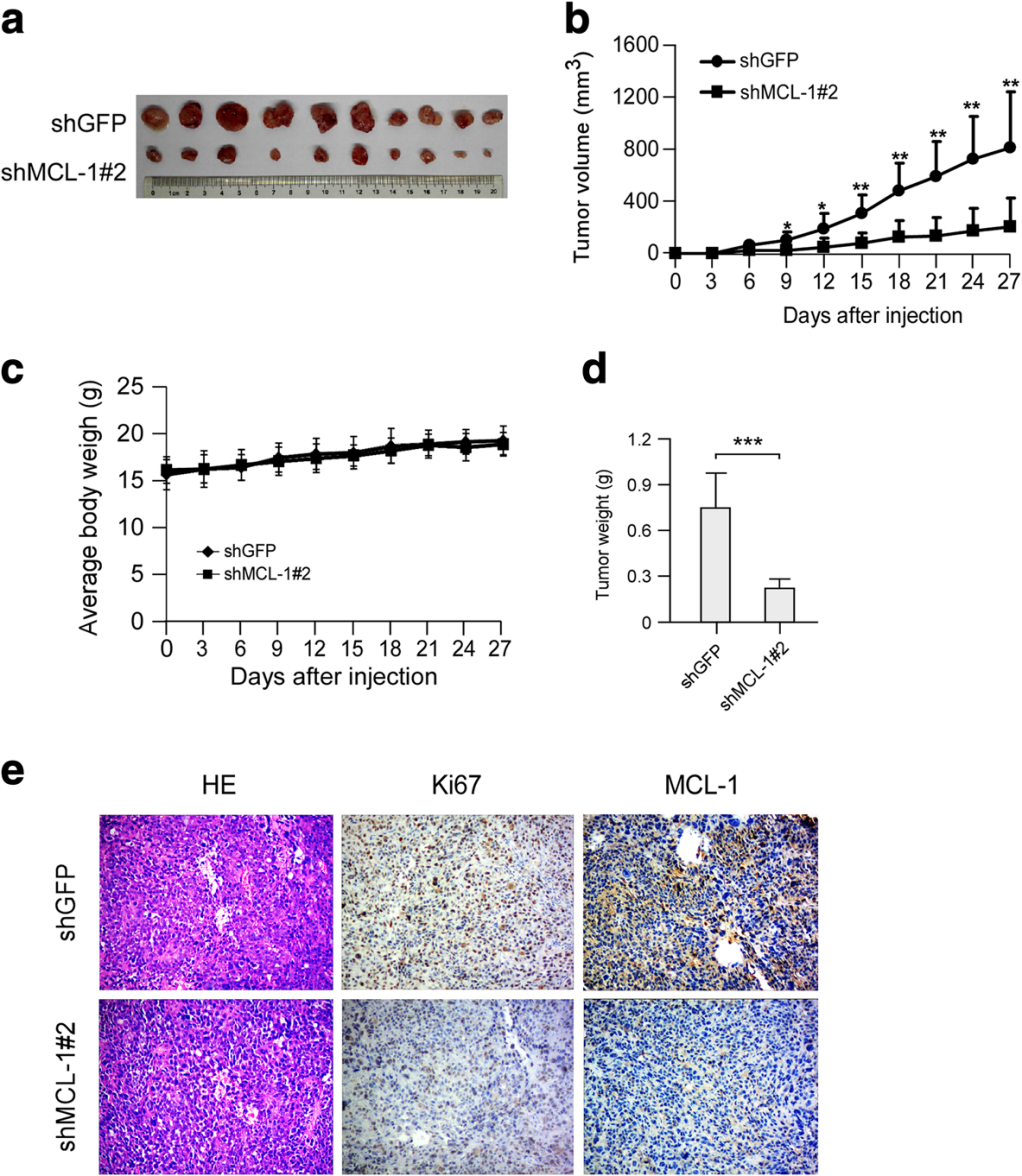
Knockdown of MCL-1 expression inhibits tumor growth in vivo. **a** Photographs of tumors dissected from mice (*n* = 10) injected with KYSE150-shGFP or KYSE150-shMCL-1#2 cells. **b** Tumor growth curve from each group was shown. **c** Average body weight of mice from each group was shown. **d** Total average tumor weight from each group was shown. Data were represented as means ± SD of each group. *p < 0.05, **p < 0.01 and ***p < 0.001, significant difference compared with the group injected with KYSE150-shGFP cells. **e** Immunohistochemical examination of Ki67 and MCL-1 in tumor sections from KYSE150-shGFP or KYSE150-shMCL-1#2 xenograft mice. Representative photographs for each antibody and each group were shown (original magnification, ×200)

We next determined whether knockdown of MCL-1 could suppress tumor growth in vivo. The KYSE150 cell line was chosen as a model for evaluation of the in vivo efficacy. The results showed that the tumor in the KYSE150-shGFP group grew faster than that in the KYSE150-shMCL-1 group **(**Fig. 4a **).** The tumor volumes were decreased in the KYSE150-shMCL-1 group **(**Fig. 4b **).** The body weights of shGFP- or shMCL-1-knockdown group were similar throughout the study **(**Fig. 4c **)**. The tumor weights were decreased in the KYSE150-shMCL-1 group **(**Fig. 4d **).** The IHC results showed that knockdown of MCL-1 dramatically decreased Ki67 staining in tumor tissue, which indicated that down-regulation of MCL-1 inhibited tumor cell proliferation in vivo **(**Fig. 4e **)**. These results suggest that blocking MCL-1 expression significantly reduces the tumorigenic properties of esophageal squamous cancer cells in vivo.

### Knockdown of MCL-1 induces apoptosis and sensitizes esophageal squamous cell carcinoma cells to cisplatin

**Fig. 5:**
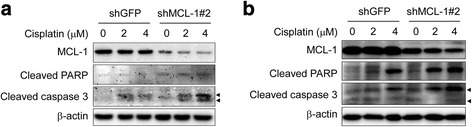
Knockdown of MCL-1 sensitizes ESCC cells to cisplatin-mediated apoptosis. **a**, **b** Stable KYSE150 (**a**) and KYSE510 (**b**) cells with MCL-1 knockdown were starved in 0.1% FBS/RPMI 1640 medium overnight and then cultured without (DMSO) or with different concentrations of cisplatin in 10% FBS/RPMI 1640 medium for 48 h. After treatment, attached and floating cells were harvested. Cleavage of caspase-3 and PARP were analyzed by Western blotting. β-actin was used as a loading control. Arrow head: 17KD or19 KD of cleaved caspase-3

**Fig. 6:**
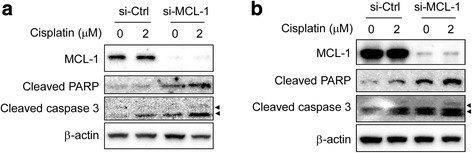
siRNA silencing of MCL-1 sensitizes cisplatin-induced apoptosis in KYSE150 and KYSE510 cell lines. **a**, **b** KYSE150 (**a**) and KYSE510 (**b**) cells were transiently transfected with si-Ctrl or si-MCL-1 for 24 h, followed by exposure to 2 μM cisplatin for 48 h. After treatment, attached and floating cells were harvested and Western blot analysis for MCL-1, cleaved PARP and cleaved caspase-3 were performed. β-actin was use as a loading control. Arrow head: 17KD or 19 KD of cleaved caspase-3

Studies have demonstrated that depletion of MCL-1 triggers apoptosis in some cancer cells, such as non-small cell lung cancer (NSCLC) cells [23] and hepatocellular carcinoma cells [5]. We then sought to determine whether knockdown of MCL-1 induced apoptosis in ESCC cells. Knockdown of MCL-1 alone slightly induced apoptosis, which was evidenced by cleavage of caspase-3 and PARP, a caspase-3 substrate, in both KYSE150 **(**Fig. 5a **,** lane 1 vs lane 4**)** and KYSE510 **(**Fig. 5b **,** lane 1 vs lane 4**)** cells. Down-regulation of MCL-1 reportedly sensitizes NSCLC cells [23] and hepatocellular carcinoma cells [5] to apoptosis induced by chemotherapeutic agents. We hypothesized that the reduction of MCL-1 level may also sensitize ESCC cells to apoptosis induced by cisplatin, a standard cytotoxic chemotherapy agent commonly used in clinical practice for ESCC patients. The results demonstrated that knockdown of MCL-1 combined treatment with cisplatin enhanced cleavage of caspase-3 and PARP in both KYSE150 **(**Fig. 5a, lane 2 vs lane 5, lane 3 vs lane 6**)** and KYSE510 **(**Fig. 5b, lane 2 vs lane 5, lane 3 vs lane 6**)** cells. Similarly, transient tansfection of an *Mcl-1* siRNA alone also induced caspase-3 and PARP cleavage **(**Fig. 6a and b **,** lane 1 vs lane 3, respectively**)** and combined treatment with 2 μM cisplatin further enhanced cleavage of caspase-3 and PARP in both KYSE150 **(**Fig. 6a, lane 2 vs lane 4**)** and KYSE510 cells **(**Fig. 6b, lane 2 vs lane 4**)**. The results indicate that suppression of MCL-1 effectively enhances the sensitivity of ESCC cells to cisplatin and suggest that manipulating antiapoptotic proteins such as MCL-1 can potentiate the anticancer effect of common chemotherapeutic drug cisplatin.

### Pharmacological inhibition of MCL-1 induces apoptotic cell death and increases cisplatin-induced apoptosis in esophageal squamous cell carcinoma cells

**Fig. 7:**
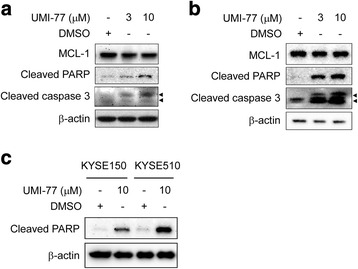
MCL-1 pharmacological inhibitor UMI-77 induces apoptosis of ESCC cells. **a**, **b** KYSE150 (**a**) and KYSE510 (**b**) cells were starved in 0.1% FBS/RPMI 1640 medium overnight and then cultured without (DMSO) or with different concentrations of UMI-77 in 10% FBS/RPMI 1640 medium for 48 h. After treatment, attached and floating cells were harvested. Cleavage of caspase-3 and PARP were analyzed by Western blotting. β-actin was used as a loading control. **c** KYSE150 and KYSE510 cells were starved in 0.1% FBS/RPMI 1640 medium overnight and then cultured without (DMSO) or with 10 μM UMI-77 in 10% FBS/RPMI 1640 medium for 48 h. After treatment, attached and floating cells were harvested. Cleavage of PARP was analyzed by Western blotting. β-actin was used as a loading control. Arrow head: 17KD or 19 KD of cleaved caspase-3

**Fig. 8:**
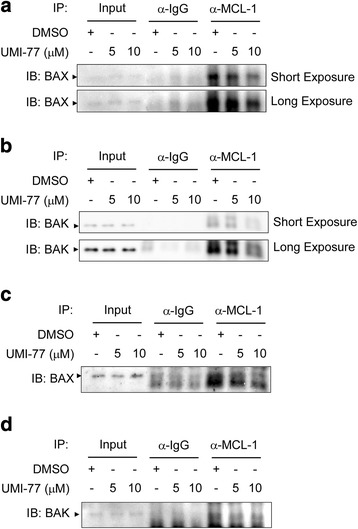
UMI-77 disrupts the endogenous protein-protein interactions of MCL-1 with BAX and BAK. **a**, **b**, **c** and d KYSE150 (**a**, **b**) and KYSE510 (**c, d**) cells were starved in 0.1% FBS/RPMI 1640 medium overnight and then cultured without (DMSO) or with different concentrations of UMI-77 in 10% FBS/RPMI 1640 medium for 48 h. After treatment, attached and floating cells were harvested. Cells were lysed and immunoprecipitated with an anti-MCL-1 or a normal IgG antibody. The immune complexes and input were analyzed by immunoblotting with a BAX (**a**, **c**) or BAK (**b**, **d**) antibody

**Fig. 9:**
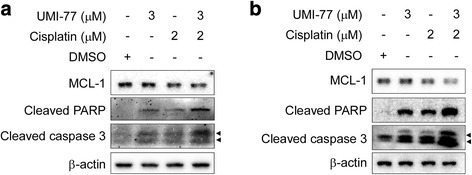
UMI-77 sensitizes ESCC cells to cisplatin-mediated apoptosis. **a**, **b** KYSE150 (**a**) and KYSE510 (**b**) were starved in 0.1% FBS/RPMI 1640 medium overnight and then exposed to the indicated concentration of UMI-77 alone, cisplatin alone, or UMI-77 in combination with cisplatin in 10% FBS/RPMI 1640 medium for 48 h. After treatment, attached and floating cells were harvested. Cleavage of caspase-3 and PARP were analyzed by Western blotting. β-actin was used as a loading control. Arrow head: 17KD or 19 KD of cleaved caspase-3

Since attenuation of MCL-1 by RNA interference lowered the threshold at which KYSE150 and KYSE510 cells undergo apoptosis and led to sensitization of these cells to undergo apoptosis triggered by cisplatin **(**Figs. 5 and 6 **)**, we predicted that a small molecule inhibitor of MCL-1 might also enhance the sensitivity of ESCC cells to this chemotherapy drug. UMI-77, a recently characterized MCL-1-specific inhibitor [24], was used to investigate the possibility. KYSE150 and KYSE510 cells were treated with UMI-77 for 48 h. Apoptosis was up-regulated in a dose dependent manner, as indicated by the presence of cleaved caspase-3 and cleaved PARP in these cell lines **(**Fig. 7a and b **)**. As expected, KYSE150 cells with higher BCL-xL level showed less sensitive to UMI-77 and less susceptible to apoptosis than did KYSE510 cells **(**Fig. 1a and b **,** Fig. 7c **)**. Co-immunoprecipitation data indicated that treatment of UMI-77 resulted in disruption of the endogenous MCL-1/BAX **(**Fig. 8a and c **)** and MCL-1/BAK **(**Fig. 8b and d **)** interactions in both KYSE150 and KYSE510 cells, which were in line with a previous report [24]. Although KYSE150 and KYSE510 cells exposed to cisplatin alone **(**Figs. 5 and 6 **)** or UMI-77 alone **(**Figs. 7 and 9 **)** underwent apoptosis compared with the DMSO control, the combination of UMI-77 with cisplatin was more potent than either agent alone in inducing cleavage of caspase-3 and PARP **(**Fig. 9a and b **)**. The results demonstrate that the combination of UMI-77 with cisplatin enhances the sensitivity of these ESCC cells to cisplatin.

## Discussion

Expression of antiapoptotic protein MCL-1 is frequently elevated in various human tumors. MCL-1 thus appears to be an attractive direct target for anticancer therapy. Pharmacological agents that cause MCL-1 depletion have been extensively investigated for anticancer therapy. Several pharmacological agents have been shown to diminish MCL-1 expression by inhibiting MCL-1 production or enhancing MCL-1 degradation. For instance, various CDK inhibitors, such as flavopiridol [25], roscovitine [26]**,** and SNS-032 [27], diminish MCL-1 levels and induce apoptosis in a variety of cell types. AZD8055, an mTORC1/2 inhibitor, reduces MCL-1 expression in *KRAS*- and *BRAF*-mutant colorectal cancer cells [28]. The USP9X inhibitor WP1130 lowers MCL-1 levels in chronic myelogenous leukemia and enhances sensitivity to apoptosis by facilitating MCL-1 degradation [29]. In addition, BH3 mimetics that block the hydrophobic BH3-binding groove of MCL-1 have been developed, which mimic the BH3 domain and therefore are able to fit into the hydrophobic pocket of MCL-1 and block its ability to bind proapoptotic proteins, inhibiting their function. These small molecules include gossypol [30], obatoclax (GX15–070) [31], sabutoclax (BI97C1) [32, 33], and BH3-M6 [34]. However, the above-mentioned small molecule BH3 mimetics are lack of selectivity for MCL-1 [35], which bind to not only MCL-1, but also other Bcl-2 family proteins, BCL-xL or/and BCL-2. Major efforts have been invested and progress has been made in developing specific inhibitors of MCL-1. Compound A-1210477, a derivative of indole-2-carboxylic acids, has been found to selectively and directly bind MCL-1, induce intrinsic apoptosis and demonstrate single agent killing of multiple myeloma and NSCLC cell lines [36]. The MCL-1-specific inhibitor UMI-77, which selectively binds to the BH3-binding groove of MCL-1, inhibits cell growth, induces apoptosis in pancreatic cancer cells and effectively inhibits BxPC-3 xenograft tumor growth [24]. Our results indicated that UMI-77 also induced apoptosis in ESCC cells when administrated as single agent **(**Figs. 7 and 9 **)** by the disruption of MCL-1 binding to BAX and BAK **(**Fig. 8 **)**. Therefore, targeting MCL-1 might qualify as a promising novel approach in ESCC therapy.

Clinically, chemotherapy is one of the most important therapeutic methods to treat numerous cancers. Cytoxic agents such as platinum (e.g. cisplatin), fluorinated pyrimidines (e.g. 5-Fu) and taxanes (e.g. paclitaxel) drugs are widely administered for chemotherapy to treat various types of cancer including ESCC, but poor response to cytoxic agent-based chemotherapy is not uncommon [37]. In view of the roles of MCL-1 in tumorigenesis, tumor progress and chemoresistance, the combinations of MCL-1 inhibitors with classical cytoxic agents have been actively investigated. It has been reported that obatoclax (GX15–070) induces apoptosis and enhances cisplatin-induced apoptosis in NSCLC cells [38]. Ren et al. [39] reported that (−)-Gossypol enhances the antitumor efficacy of cisplatin through inhibition of APE1 repair and redox activity in non-small cell lung cancer. Furthermore, synergistic antitumor effects have been observed when MCL-1 inhibitors combined not only with cytoxic drugs but also with other chemotherapeutic agents. For instance, MCL-1 inhibitor sabutoclax (BI97C1) and COX-2 inhibitor celecoxib synergistically inhibits the growth of oral squamous cell carcinomas cells both in vitro and in vivo [40]. Sabutoclax also reportedly synergizes with minocycline to induce growth arrest and apoptosis in pancreatic cancer cells [41]. Our results demonstrated that UMI-77 synergistically enhanced cisplatin-induced apoptosis in both KYSE150 and KYSE510 cells **(**Fig. 9 **)**. Since MCL-1 was overexpressed in more than 60% of ESCC patient samples **(**Table 1 **)**, it might contribute to poor response to chemotherapy in some of the ESCC patients. The enhanced apoptosis when cisplatin in combination with MCL-1 knockdown **(**Figs. 5 and 6 **)** or with MCL-1 inhibitor UMI-77 **(**Fig. 9 **)** further suggested that the combination of cisplatin with other therapies that modulate MCL-1 could be exploited as a plausible strategy to enhance therapeutic efficacy for ESCCs.

Our results indicated that, among the ESCC cell lines evaluated, KYSE510 cell line with the highest level of MCL-1 and the lowest level of BCL-xL exhibited high susceptibility to UMI-77-induced apopotosis. However, KYSE150 cell line expressing similar level of MCL-1 and higher level of BCL-xL displayed less sensitivity to UMI-77 treatment compared with KYSE510 cell line **(**Fig. 1a and b **,** Fig 7c **)**. As the survival of most tumors is not dependent on a single antiapoptotic Bcl-2 protein, strategies that combination of MCL-1 inhibition with inhibitors targeting different Bcl-2 family members would be more successful than therapies targeting only a single antiapoptotic Bcl-2 family protein. For instance, down-regulation of MCL-1 enhances cell-killing abilities of ABT-737 and ABT-263, which both inhibit BCL-2 and BCL-xL but not MCL-1 [42]. MCL-1 down-regulation by CDK inhibitor roscovitine or *Mcl-1*-shRNA dramatically increases ABT-737 lethality in human leukemia cells [43]. Faber et al. [44] reported that the combination of ABT-263 and AZD8055, an mTORC1/2 inhibitor that reduced MCL-1 protein levels, potently suppresses tumor progression across a variety of preclinical small cell lung cancer experimental models. Potent and selective small-molecule MCL-1 inhibitors A-1210477 synergizes with the BCL-2 and BCL-xL inhibitor ABT-263 to kill a variety of cancer cell lines [36]. In some cancer types, especially for those cell lines that rely on multiple Bcl-2 family members for survival, efficient treatment will more commonly require either a pan-Bcl-2 family protein inhibitor or a combination of inhibitors that neutralises the different Bcl-2 family members, which could be a rational approach in treating tumors.

Mechanistically, apoptosis induction by UMI-77 is BAX/BAK-dependent, preceded by disrupting disruption of MCL-1 binding to BAX and BAK **(**Fig. 8 **)**. Although we did not investigate the activity of UMI-77 in animal tumor models, previous study by Abulwerdi et al. [24] have shown that UMI-77 is well-tolerated and inhibits the growth of pancreatic tumor xenografts with no apparent toxicity in normal mouse tissues. This study revealed the presence of TUNEL-positive apoptotic cells in tumors collected from UMI-77-treated animals, further supporting our observations that induction of apoptosis is, at least in part, a mechanism of action of UMI-77. Since the in vitro data in our present study demonstrated the efficacy of UMI-77 as a chemosensitizing agent in ESCC cells, it will be important in future studies to determine the anti-tumor effects as well as toxicity of the combination of UMI-77 with cytotoxic drugs or other chemotherapeutic agents in animal models.

It has been well examined that the multidomain proapoptotic proteins BAK and BAX are executors of the mitochondrial pathway of apoptosis whose activation can be prevented by antiapoptotic Bcl-2 family proteins such as MCL-1 and BCL-xL [20, 45]. Although BAX and BAK seem in most circumstances to be functionally equivalent, substantial differences exist. BAX is largely cytosolic, whereas BAK resides in complexes on the outer membrane of mitochondria and on the endoplasmic reticulum of healthy cells. Nevertheless, the activation of BAX and BAK appears similar [46]. On receipt of cytotoxic signals, both BAX and BAK change conformation, and BAX translocates to the mitochondrial outer membrane, where both BAX and BAK then form homo-oligomers that can associate, leading to membrane permeabilization [20, 46]. In some cell types, such as chronic myelogenous leukemia [47] and multiple myeloma [48], the forced reduction of MCL-1 permits BAK oligomerization, activation and is sufficient enough to induce apoptotic cell death. However, some types of cells require a second signal such as genotoxic stress to induce apoptosis [49]. This difference may be accounted for the different level of BCL-xL, because BCL-xL may replace MCL-1 in its suppression of BAK activation [50]. In the case of BAX, it does not co-immunoprecipitate with MCL-1 or BCL-xL in HeLa cells [20]. However, the apoptosis induced by MCL-1 suppression was partially mediated through BAX in rheumatoid arthritis synovial fibroblasts [51] and in pancreatic cancer cells [24]. Our results indicated that KYSE510 cells expressed lower BCL-xL protein level than KYSE150 cells **(**Fig. 1a and b **),** which accompanied by a stronger response to cisplatin- or UMI-77-induced apopotosis than did KYSE150 cells **(**Figs. 5, 6, 7 and 9 **)**. The detail mechanism by which BCL-xL replaces MCL-1 and suppresses BAK activation and whether coordinately targeting both MCL-1/BAK axis and BCL-xL/BAK axis heighten the sensitivity of ESCC cells to cisplatin-induced apoptosis need to be further investigated.

## Conclusions

Our results suggests that MCL-1 contributes to the development of ESCC and provide insights into the potential role of MCL-1 as a therapeutic target in ESCC chemotherapy and show that antagonizing MCL-1 function with RNAi-mediated knockdown or small molecule MCL-1 inhibitor UMI-77 sensitizes ESCC cells to cisplatin-induced apoptosis. Our results suggest that targeting MCL-1 in combination with other chemotherapic agents might be a plausible therapeutic strategy to enhance the treatment efficacy for ESCCs.

## Abbreviations

Co-IP: Co-immunoprecipitation
ESCC: Esophageal squamous cell carcinoma
IHC: Immunohistochemical
MCL-1: Myeloid cell leukemia 1
NSCLC: Non-small cell lung cancer
pTNM: pathologic tumor-node-metastasis
RT-PCR: Reverse transcription-RCR
shRNAs: short hairpin RNAs

## References

[1] Ferlay J, Shin HR, Bray F, Forman D, Mathers C, Parkin DM (2010). Estimates of worldwide burden of cancer in 2008: GLOBOCAN 2008. Int J Cancer.

[2] Akgul C (2009). Mcl-1 is a potential therapeutic target in multiple types of cancer. Cell Mol Life Sci.

[3] Lee WS, Park YL, Kim N, Oh HH, Son DJ, Kim MY, Oak CY, Chung CY, Park HC, Kim JS (2015). Myeloid cell leukemia-1 regulates the cell growth and predicts prognosis in gastric cancer. Int J Oncol.

[4] Craig RW (2002). MCL1 provides a window on the role of the BCL2 family in cell proliferation, differentiation and tumorigenesis. Leukemia.

[5] Sieghart W, Losert D, Strommer S, Cejka D, Schmid K, Rasoul-Rockenschaub S, Bodingbauer M, Crevenna R, Monia BP, Peck-Radosavljevic M (2006). Mcl-1 overexpression in hepatocellular carcinoma: a potential target for antisense therapy. J Hepatol.

[6] Miyamoto Y, Hosotani R, Wada M, Lee JU, Koshiba T, Fujimoto K, Tsuji S, Nakajima S, Doi R, Kato M (1999). Immunohistochemical analysis of Bcl-2, Bax, Bcl-X, and Mcl-1 expression in pancreatic cancers. Oncology.

[7] Dash R, Richards JE, Su ZZ, Bhutia SK, Azab B, Rahmani M, Dasmahapatra G, Yacoub A, Dent P, Dmitriev IP (2010). Mechanism by which Mcl-1 regulates cancer-specific apoptosis triggered by mda-7/IL-24, an IL-10-related cytokine. Cancer Res.

[8] Whitsett TG, Mathews IT, Cardone MH, Lena RJ, Pierceall WE, Bittner M, Sima C, LoBello J, Weiss GJ, Tran NL (2014). Mcl-1 mediates TWEAK/Fn14-induced non-small cell lung cancer survival and therapeutic response. Mol Cancer Res.

[9] Liu H, Yang J, Yuan Y, Xia Z, Chen M, Xie L, Ma X, Wang J, Ouyang S, Wu Q (2014). Regulation of Mcl-1 by constitutive activation of NF-kappaB contributes to cell viability in human esophageal squamous cell carcinoma cells. BMC Cancer.

[10] Leu CM, Chang C, Hu C (2000). Epidermal growth factor (EGF) suppresses staurosporine-induced apoptosis by inducing mcl-1 via the mitogen-activated protein kinase pathway. Oncogene.

[11] Feng YB, Lin DC, Shi ZZ, Wang XC, Shen XM, Zhang Y, Du XL, Luo ML, Xu X, Han YL (2009). Overexpression of PLK1 is associated with poor survival by inhibiting apoptosis via enhancement of survivin level in esophageal squamous cell carcinoma. Int J Cancer.

[12] Ding Q, He X, Hsu JM, Xia W, Chen CT, Li LY, Lee DF, Liu JC, Zhong Q, Wang X (2007). Degradation of Mcl-1 by beta-TrCP mediates glycogen synthase kinase 3-induced tumor suppression and chemosensitization. Mol Cell Biol.

[13] Yin W, Nie Y, Zhang Z, Xie L (2015). He X: miR-193b acts as a cisplatin sensitizer via the caspase-3-dependent pathway in HCC chemotherapy. Oncol Rep.

[14] Hu J, Xu C, Cheng B, Jin L, Li J, Gong Y, Lin W, Pan Z, Pan C (2016). Imperatorin acts as a cisplatin sensitizer via downregulating Mcl-1 expression in HCC chemotherapy. Tumour Biol.

[15] Tanaka N, Patel AA, Wang J, Frederick MJ, Kalu NN, Zhao M, Fitzgerald AL, Xie TX, Silver NL, Caulin C (2015). Wee-1 Kinase inhibition sensitizes high-risk HPV+ HNSCC to apoptosis accompanied by Downregulation of MCl-1 and XIAP Antiapoptotic proteins. Clin Cancer Res.

[16] Akagi H, Higuchi H, Sumimoto H, Igarashi T, Kabashima A, Mizuguchi H, Izumiya M, Sakai G, Adachi M, Funakoshi S (2013). Suppression of myeloid cell leukemia-1 (Mcl-1) enhances chemotherapy-associated apoptosis in gastric cancer cells. Gastric Cancer.

[17] Stoner GD, Kaighn ME, Reddel RR, Resau JH, Bowman D, Naito Z, Matsukura N, You M, Galati AJ, Harris CC (1991). Establishment and characterization of SV40 T-antigen immortalized human esophageal epithelial cells. Cancer Res.

[18] Liu H, Hwang J, Li W, Choi TW, Liu K, Huang Z, Jang JH, Thimmegowda NR, Lee KW, Ryoo IJ (2014). A derivative of chrysin suppresses two-stage skin carcinogenesis by inhibiting mitogen- and stress-activated kinase 1. Cancer Prev Res (Phila).

[19] Liu H, Liu K, Huang Z, Park CM, Thimmegowda NR, Jang JH, Ryoo IJ, He L, Kim SO, Oi N (2013). A chrysin derivative suppresses skin cancer growth by inhibiting cyclin-dependent kinases. J Biol Chem.

[20] Willis SN, Chen L, Dewson G, Wei A, Naik E, Fletcher JI, Adams JM, Huang DC (2005). Proapoptotic Bak is sequestered by Mcl-1 and Bcl-xL, but not Bcl-2, until displaced by BH3-only proteins. Genes Dev.

[21] van Delft MF, Huang DC (2006). How the Bcl-2 family of proteins interact to regulate apoptosis. Cell Res.

[22] Adams JM, Cory S (2007). The Bcl-2 apoptotic switch in cancer development and therapy. Oncogene.

[23] Song L, Coppola D, Livingston S, Cress D, Haura EB (2005). Mcl-1 regulates survival and sensitivity to diverse apoptotic stimuli in human non-small cell lung cancer cells. Cancer Biol Ther.

[24] Abulwerdi F, Liao C, Liu M, Azmi AS, Aboukameel A, Mady AS, Gulappa T, Cierpicki T, Owens S, Zhang T (2014). A novel small-molecule inhibitor of mcl-1 blocks pancreatic cancer growth in vitro and in vivo. Mol Cancer Ther.

[25] Kitada S, Zapata JM, Andreeff M, Reed JC (2000). Protein kinase inhibitors flavopiridol and 7-hydroxy-staurosporine down-regulate antiapoptosis proteins in B-cell chronic lymphocytic leukemia. Blood.

[26] Hahntow IN, Schneller F, Oelsner M, Weick K, Ringshausen I, Fend F, Peschel C, Decker T (2004). Cyclin-dependent kinase inhibitor Roscovitine induces apoptosis in chronic lymphocytic leukemia cells. Leukemia.

[27] Chen R, Wierda WG, Chubb S, Hawtin RE, Fox JA, Keating MJ, Gandhi V, Plunkett W (2009). Mechanism of action of SNS-032, a novel cyclin-dependent kinase inhibitor, in chronic lymphocytic leukemia. Blood.

[28] Faber AC, Coffee EM, Costa C, Dastur A, Ebi H, Hata AN, Yeo AT, Edelman EJ, Song Y, Tam AT (2013). mTOR inhibition specifically sensitizes colorectal cancers with KRAS or BRAF mutations to BCL-2/BCL-XL inhibition by suppressing MCL-1. Cancer Discov.

[29] Sun H, Kapuria V, Peterson LF, Fang D, Bornmann WG, Bartholomeusz G, Talpaz M, Donato NJ (2011). Bcr-Abl ubiquitination and Usp9x inhibition block kinase signaling and promote CML cell apoptosis. Blood.

[30] Wei J, Kitada S, Rega MF, Emdadi A, Yuan H, Cellitti J, Stebbins JL, Zhai D, Sun J, Yang L (2009). Apogossypol derivatives as antagonists of antiapoptotic Bcl-2 family proteins. Mol Cancer Ther.

[31] Nguyen M, Marcellus RC, Roulston A, Watson M, Serfass L, Murthy Madiraju SR, Goulet D, Viallet J, Belec L, Billot X (2007). Small molecule obatoclax (GX15-070) antagonizes MCL-1 and overcomes MCL-1-mediated resistance to apoptosis. Proc Natl Acad Sci U S A.

[32] Wei J, Stebbins JL, Kitada S, Dash R, Placzek W, Rega MF, Wu B, Cellitti J, Zhai D, Yang L (2010). BI-97C1, an optically pure Apogossypol derivative as pan-active inhibitor of antiapoptotic B-cell lymphoma/leukemia-2 (Bcl-2) family proteins. J Med Chem.

[33] Dash R, Azab B, Quinn BA, Shen X, Wang XY, Das SK, Rahmani M, Wei J, Hedvat M, Dent P (2011). Apogossypol derivative BI-97C1 (Sabutoclax) targeting Mcl-1 sensitizes prostate cancer cells to mda-7/IL-24-mediated toxicity. Proc Natl Acad Sci U S A.

[34] Kazi A, Sun J, Doi K, Sung SS, Takahashi Y, Yin H, Rodriguez JM, Becerril J, Berndt N, Hamilton AD (2011). The BH3 alpha-helical mimic BH3-M6 disrupts Bcl-X(L), Bcl-2, and MCL-1 protein-protein interactions with Bax, Bak, bad, or Bim and induces apoptosis in a Bax- and Bim-dependent manner. J Biol Chem.

[35] Quinn BA, Dash R, Azab B, Sarkar S, Das SK, Kumar S, Oyesanya RA, Dasgupta S, Dent P, Grant S (2011). Targeting Mcl-1 for the therapy of cancer. Expert Opin Investig Drugs.

[36] Leverson JD, Zhang H, Chen J, Tahir SK, Phillips DC, Xue J, Nimmer P, Jin S, Smith M, Xiao Y (2015). Potent and selective small-molecule MCL-1 inhibitors demonstrate on-target cancer cell killing activity as single agents and in combination with ABT-263 (navitoclax). Cell Death Dis.

[37] Ilson DH (2008). Esophageal cancer chemotherapy: recent advances. Gastrointest Cancer Res.

[38] Li J, Viallet J, Haura EB (2008). A small molecule pan-Bcl-2 family inhibitor, GX15-070, induces apoptosis and enhances cisplatin-induced apoptosis in non-small cell lung cancer cells. Cancer Chemother Pharmacol.

[39] Ren T, Shan J, Li M, Qing Y, Qian C, Wang G, Li Q, Lu G, Li C, Peng Y (2015). Small-molecule BH3 mimetic and pan-Bcl-2 inhibitor AT-101 enhances the antitumor efficacy of cisplatin through inhibition of APE1 repair and redox activity in non-small-cell lung cancer. Drug Des Devel Ther.

[40] Maji S, Samal SK, Pattanaik L, Panda S, Quinn BA, Das SK, Sarkar D, Pellecchia M, Fisher PB, Dash R (2015). Mcl-1 is an important therapeutic target for oral squamous cell carcinomas. Oncotarget.

[41] Quinn BA, Dash R, Sarkar S, Azab B, Bhoopathi P, Das SK, Emdad L, Wei J, Pellecchia M, Sarkar D (2015). Pancreatic cancer combination therapy using a BH3 mimetic and a synthetic tetracycline. Cancer Res.

[42] Varadarajan S, Vogler M, Butterworth M, Dinsdale D, Walensky LD, Cohen GM (2013). Evaluation and critical assessment of putative MCL-1 inhibitors. Cell Death Differ.

[43] Chen S, Dai Y, Harada H, Dent P, Grant S (2007). Mcl-1 down-regulation potentiates ABT-737 lethality by cooperatively inducing Bak activation and Bax translocation. Cancer Res.

[44] Faber AC, Farago AF, Costa C, Dastur A, Gomez-Caraballo M, Robbins R, Wagner BL, Rideout WM, Jakubik CT, Ham J (2015). Assessment of ABT-263 activity across a cancer cell line collection leads to a potent combination therapy for small-cell lung cancer. Proc Natl Acad Sci U S A.

[45] Green DR, Evan GI (2002). A matter of life and death. Cancer Cell.

[46] Gelinas C, White E (2005). BH3-only proteins in control: specificity regulates MCL-1 and BAK-mediated apoptosis. Genes Dev.

[47] Aichberger KJ, Mayerhofer M, Krauth MT, Skvara H, Florian S, Sonneck K, Akgul C, Derdak S, Pickl WF, Wacheck V (2005). Identification of mcl-1 as a BCR/ABL-dependent target in chronic myeloid leukemia (CML): evidence for cooperative antileukemic effects of imatinib and mcl-1 antisense oligonucleotides. Blood.

[48] Zhang B, Gojo I, Fenton RG (2002). Myeloid cell factor-1 is a critical survival factor for multiple myeloma. Blood.

[49] Nijhawan D, Fang M, Traer E, Zhong Q, Gao W, Du F, Wang X (2003). Elimination of Mcl-1 is required for the initiation of apoptosis following ultraviolet irradiation. Genes Dev.

[50] Mandelin AM, Pope RM (2007). Myeloid cell leukemia-1 as a therapeutic target. Expert Opin Ther Targets.

[51] Liu H, Eksarko P, Temkin V, Haines GK, Perlman H, Koch AE, Thimmapaya B, Pope RM (2005). Mcl-1 is essential for the survival of synovial fibroblasts in rheumatoid arthritis. J Immunol.

